# Immunosuppressive Drugs in Early Systemic Sclerosis and Prevention of Damage Accrual

**DOI:** 10.1002/acr.25467

**Published:** 2025-02-02

**Authors:** Murray Baron, Mandana Nikpour, Dylan Hansen, Susanna Proudman, Wendy Stevens, the Australian Scleroderma Interest Group and the Canadian Scleroderma Research Group, Joanne Sahhar, Nava Ferdowsi, Kathleen Morrisroe, Laura Ross, Gene Siew Ngian, Jennifer Walker, Janet Roddy, Lauren Host, Mohammed Osman, Peter Docherty, Paul Fortin, Marvin Jacob Fritzler, Genevieve Gyger, Alena Ikic, Niall Jones, Elzbieta Kaminska, Maggie Larche, Sophie Ligier, Ada Man, Ariel Masetto, Janet Pope, David Robinson, Tatiana Sofia Rodriguez‐Reyna, Mianbo Wang

**Affiliations:** ^1^ Jewish General Hospital, McGill University Montreal Quebec Canada; ^2^ The University of Sydney and Royal Prince Alfred Hospital Camperdown New South Wales Australia; ^3^ The University of Sydney Camperdown New South Wales Australia; ^4^ Royal Adelaide Hospital Adelaide South Australia Australia; ^5^ St. Vincent's Hospital Melbourne Fitzroy Victoria Australia; ^6^ Lady Davis Institute for Medical Research Montreal Quebec Canada

## Abstract

**Objective:**

Organ damage in patients with systemic sclerosis (SSc) in individual organs such as the lungs may be prevented by receiving immunosuppressive drugs (ISs). A new measure of global organ damage, the Scleroderma Clinical Trials Consortium Damage Index (SCTC‐DI), has allowed us to investigate whether receiving ISs may reduce global organ damage accrual in patients with early SSc.

**Methods:**

This was a retrospective study of patients with two or less years of disease duration in Canadian and Australian cohorts with SSc. Patients with either limited cutaneous SSc (lcSSc) or diffuse cutaneous SSc (dcSSc) were observed separately and divided into groups who were either ever or never exposed to ISs. The SCTC‐DI was the outcome, and inverse probability of treatment weighting (IPTW) was used to balance the study groups and to fit a marginal structural generalized estimating equation model.

**Results:**

In the cohort with lcSSc, there were 210 patients, of whom 34% were exposed to ISs at some time. Exposure to ISs was associated with lower damage scores. In the cohort with dcSSc, there were 192 patients, of whom 76% were exposed to ISs at some time. Exposure to ISs was not associated with damage scores.

**Conclusion:**

In this retrospective observational cohort study, using IPTW to adjust for confounders, we found a protective effect of receiving ISs on damage accrual in patients with lcSSc. We were unable to determine such an effect in patients with dcSSc, but unknown confounders may have been present, and prospective studies of patients with dcSSc receiving ISs should include the SCTC‐DI to determine the possible effect of ISs on damage accrual.

## INTRODUCTION

Systemic sclerosis (SSc) is an autoimmune disorder characterized by vasculopathy, immunologic abnormalities, and fibrosis, affecting both skin and visceral organs. Although there is no cure, accumulating evidence shows that immunosuppression may be effective in stabilizing and perhaps improving manifestations of SSc, including skin thickening and interstitial lung disease (ILD).^1^, ^2^, ^3^, ^4^, ^5^, ^6^, ^7^, ^8^, ^9^, ^10^, ^11^ Current recommendations support prescribing immunosuppressive drugs (ISs) for treatment of these manifestations of SSc.^9^

**SIGNIFICANCE & INNOVATIONS**

- The effect of receiving immunosuppressive drugs in patients with systemic sclerosis (SSc) on global damage has not been assessed before.
- Using the Scleroderma Clinical Trials Consortium Damage Index and data from Canada and Australia, we assessed the effect of receiving immunosuppressive drugs in patients with SSc on global damage.
- In patients with early disease, we have found that receiving immunosuppressive drugs reduces damage accrual in patients with limited cutaneous SSc.
- More research is needed to assess the effect of receiving immunosuppressive drugs on global damage in patients with early diffuse cutaneous SSc.

Organ damage is common and an important cause of mortality and morbidity in patients with SSc, and irreversible organ damage is accrued very early in the disease course, with 40% of patients having damage in one or more organ systems within two years of disease onset.^12^, ^13^, ^14^, ^15^ The relationship between immune abnormalities and organ damage is not completely clear, but studies have suggested that immunologic changes can ultimately lead to tissue fibrosis, a hallmark of disease damage in SSc.^16^, ^17^, ^18^, ^19^, ^20^ It was previously difficult to quantitate organ damage accrual in SSc, but the development and validation of the Scleroderma Clinical Trials Consortium Damage Index (SCTC‐DI) has opened the door to studies of damage accrual in this disease.^12^, ^13^, ^14^

The objective of this study was to assess the relationship between receiving ISs and the development of organ damage over time in a longitudinal observational cohort of patients with SSc. The ISs assessed were methotrexate (MTX), cyclophosphamide (CYC), azathioprine (AZA), and mycophenolate (MPA). Our hypothesis was that receiving ISs would prevent the development of damage compared to not receiving such medications. To adjust for imbalance in measured confounders between groups who were and were not exposed, inverse probability of treatment weighting (IPTW) was used to balance the characteristics of the two patient groups.

## PATIENTS AND METHODS

### Study populations

Participants were recruited from the Canadian Scleroderma Research Group (CSRG) registry and from the Australian Scleroderma Cohort Study (ASCS). The CSRG recruits and follows patients from 15 centers in Canada and Mexico. These centers see local and regional referrals. All patients must have a diagnosis of SSc (confirmed by an experienced rheumatologist), be ≥18 years of age, provide informed consent, and be fluent in English, French, or Spanish. Over 98% of the cohort meets the 2013 American College of Rheumatology (ACR)/EULAR classification criteria for SSc.^21^ The ASCS is an Australian multicenter cohort study of risk and prognostic factors in SSc. The ASCS and CSRG have been approved by all human research ethics committees of participating sites. For a complete list of members of the Australian Scleroderma Interest Group and the CSRG, who collected data for this paper and reviewed the manuscript, see Appendices A and B. All participants met ACR/EULAR criteria for SSc.^21^ Written informed consent was obtained from all participants at recruitment. We included only those participants recruited within two years of onset of the first non‐Raynaud manifestation of SSc. Patients with diffuse cutaneous SSc (dcSSc) were defined by skin thickening proximal to the elbows or knees and/or trunk at any time. Ethics committee approval for this study was obtained at the Jewish General Hospital, Montreal, Canada (approval number 2019‐1597) and at all participating CSRG study sites. Ethics approvals for Australian data were obtained from St. Vincent's Hospital Melbourne (approval number LRR 012/21) and all participating ASCS sites.

### Exposure

Medication exposure was recorded yearly by study physicians and coded as current, past, or never. Patients were recorded at each visit as being exposed or not to ISs (MTX, CYC, AZA, or MPA). At the baseline visit either no IS, current IS, or IS not current but before the visit exposure was recorded. In our analysis, “immunosuppressants before baseline” refers to ISs not received at the baseline visit but only before that visit.

### SCTC‐DI

The SCTC‐DI measures global irreversible damage in patients with SSc and was developed to be highly correlated with mortality and morbidity (measured by the 36‐item short‐form (SF‐36) survey).^22^ Validated and published in 2019, its development was an international collaboration among 22 experts with input from patient partners using a combined approach of consensus and data‐driven methods. The index is composed of 23 differently weighted items in several organ systems (musculoskeletal, skin, vascular, gastrointestinal, respiratory, cardiovascular, and renal). Low, medium, and high SCTC‐DI scores are defined as <5, 6 to 12, and ≥13, respectively, with a maximum score of 55. For the definition of the individual components of the SCTC‐DI, please refer to the original paper.^22^

In this study, the SCTC‐DI was calculated using registry data, but three items (calcinosis complicated by infection or requiring surgery, gastric antral vascular ectasia, and right ventricular dysfunction) were removed because they were not collected in the CSRG database, and one item (small‐joint contractures) was removed due to missing data (>20% of visits). The maximum SCTC‐DI score possible was therefore 42. Scores are always whole integers, as are changes in scores over time.

To score the SCTC‐DI for lung disease, high‐resolution computed tomography (HRCT) scans were assessed by local radiologists, and there was no central reading. CT scans were not necessarily done according to any protocol but were done at the request of the treating physician. Once a damage score was assigned, it is carried forward until something occurs to increase it. In the case of the lung, an increase in score would occur if another CT scan showed an increase of ILD on CT to ≥20% or a drop in forced vital capacity (FVC) to <70% predicted. The damage score is calculated from the data acquired at each patient visit. The SCTC‐DI is scored such that patients cannot improve; they can only stay the same or worsen, so unless there is worsening in a particular organ, the score remains the same as the previous visit.

### Definition of variables

Disease duration was defined from the onset of the first non‐Raynaud phenomenon symptom to the index visit (Table 1). Smoking status was classified as either never smoker or past and/or current smoker. Skin involvement was assessed using the modified Rodnan skin score (mRSS), which ranges from 0 (no involvement) to 3 (severe thickening) in 17 areas (score range 0–51). FVC percentage was extracted from pulmonary function tests. The presence of ILD was determined using a published clinical decision rule.^23^ Using this rule, ILD was considered present if an HRCT scan of the lung was interpreted by an experienced radiologist as showing ILD or, in the case of no HRCT being available, if either a chest x‐ray was reported as showing either increased interstitial markings (not thought to be due to congestive heart failure) or fibrosis and/or if a study physician reported the presence of typical “Velcro‐like crackles” on physical examination. Physical function was assessed using the Health Assessment Questionnaire Disability Index, which is scored from 0 (no disability) to 3 (severe disability). Patient and physician global assessment scores were rated 0 to 10 (no disease to very severe disease) on numeric rating scales. For patient assessment scores, patients were asked, “In the past week, how was your overall health?” The physician global severity question asked, “How would you rate the patient's overall health for the past week?” Other covariates recorded at the index visit included physician reports of inflammatory myositis, arthritis, digital ulcers, and prior scleroderma renal crisis. Pulmonary hypertension was defined as an estimated systolic pulmonary artery pressure ≥45 mm Hg measured using the Doppler flow measurement of the tricuspid regurgitant jet on cardiac echocardiography (used as a noninvasive screening tool for pulmonary hypertension).^24^ Antinuclear antibody was detected by immunofluorescence, and other autoantibodies were detected by line immunoassay (Euroimmun).

**Table 1:** Baseline characteristics of limited cutaneous scleroderma patients (lcSSc) stratified according to exposure to immunosuppression at baseline or during follow‐up (*N* = 210)*

	Ever exposed to IS at any visit (*n* = 71)		Never exposed to IS at any visits (*n* = 139)		*P*‐values
		Missing data		Missing data	
		*N*		*N*	
Age, years (mean ± SD)	55.1 ± 11.8		56.4 ± 13.7		0.509
Female, N (%)	60 (84.5%)		121 (87.1%)		0.613
Caucasian, N (%)	63 (92.6%)	3	113 (83.7%)	4	0.077
Education (>high school), *N* (%)	31 (45.6%)	3	60 (46.5%)	10	0.902
Smoking in the past or currently, *N* (%)	39 (54.9%)		71 (51.1%)		0.597
Disease duration, years (mean ± SD)	1.1 ± 0.5		1.0 ± 0.5		0.517
mRSS (0−51) (mean ± SD)	5.9 ± 4.3	1	4.4 ± 4.2	1	0.003
Interstitial lung disease, *N* (%)	23 (32.4%)		27 (19.4%)		0.037
FVC, % predicted (mean ± SD)	93.8 ± 20.8	0	97.5 ± 20.2	1	0.214
DLCO, % predicted (mean ± SD)	69.0 ± 20.6	4	73.3 ± 22.6	9	0.192
Tendon friction rubs, *N* (%)	4 (5.6%)	0	3 (2.2%)	2	0.233
Inflammatory arthritis, N (%)	34 (48.6%)	1	30 (22.2%)	4	<0.001
Autoantibodies					
Anti‐centromere, *N* (%)	20 (30.0%)	2	79 (59.4%)	6	<.001
Anti‐topoisomerase, *N* (%)	23 (33.3%)	2	13 (9.7%)	5	<.001
Anti‐RNA polymerase III, *N* (%)	6 (9.1%)	5	9 (7.3%)	16	0.667
C‐reactive protein, mg/L (median, IQR)	4 (2−7)	6	3.7( 1.4−6.4)	9	0.109
Damage score at baseline (median, IQR)	3 (2−5)		3 (1−5)		0.139
CSRG patients, *N* (%)	32 (45.1%)		56 (40.3%)		0.506
Any Immunosuppressants prior to but not at baseline, *N* (%)	6 (8.5%)		4 (2.9%)		0.147

* CSRG, Canadian Scleroderma Research Group; DLCO, diffusing capacity of carbon monoxide; FVC, forced vital capacity; IS, immunosuppressives; mRSS, modified Rodnan skin score.

### Statistical analysis

Because of the very different trajectories of damage in limited cutaneous SSc (lcSSc) and dcSSc,^12^, ^13^ and because exposure to ISs was so different in the subsets of patients with cutaneous SSc (145 of 192 patients or 76% in the subset with dcSSc and 71 of 210 or 34% in the subset with lcSSc), we analyzed these two subsets separately. Descriptive statistics were used to summarize baseline demographic and clinical characteristics of the patients who were and were not exposed to ISs. Continuous variables are presented as mean ± SD, and categorical variables are presented as counts and percentages. Student's *t*‐test and Wilcoxon–Mann–Whitney U test were used to compare continuous variables. Chi‐square test and Fisher exact test were used for categorical variables.

Due to the inherent differences between patients who were and were not exposed to ISs in an observational study, IPTW was used to balance the study groups and to fit a marginal structural generalized estimating equation (GEE) model.^25^ Each in‐person visit was treated as an observation. Propensity scores representing the probability of being exposed at a given visit were calculated using a pooled logistic regression adjusting for baseline covariates (sex, age, disease duration, exposure to immunosuppression before baseline visit, FVC at baseline, damage score at baseline visit) and time‐varying covariates (mRSS and presence of arthritis) at each annual visit. These time‐varying variables not only affect treatment but also associate with the outcome, and they also may be affected by the previous treatment. They are potentially time‐varying confounding, which is important to adjust for as time‐varying variables in the propensity score model for the IPTW.

To evaluate for residual differences in baseline covariates between the two statistically matched groups, we calculated the standardized mean difference (SMD) of each disease variable. The SMD is the difference in means of a covariate across the treatment groups divided by the SD in the treated group. It is the most commonly used statistic to examine the balance of covariate distribution between treatment groups.^26^, ^27^ Because the SMD is independent of the unit of measurement, it allows comparison between variables with different units of measurement. A standardized difference of ≤0.1 represents meaningful balance.^26^, ^27^

All models include time‐varying variables. We have adjusted and unadjusted models for weights. The adjusted model was used to account for the time‐varying variables and generated the denominator of the weights. The unadjusted model was used to generate the numerator of the weights, which created more stabilized weights. These stabilized weights were then used in a weighted GEE model to estimate the parameters of the marginal structural model. This model was conditional on exposure to ISs at the given visit and adjusted for sex, age, disease duration, diffuse subset, exposure to immunosuppression before baseline visit, and damage score at baseline visit. The cohort (CSRG vs ASCS) was also adjusted in the GEE outcome model. Because the damage scores are always increasing, we used the change of damage scores between two visits (eg, damage score at current visit minus damage score at previous visit) as the outcome at each visit instead of the raw scores. Because the most common antibodies in patients with lcSSc were anti‐centromere and anti‐topoisomerase and in patients with dcSSc were anti–RNA polymerase III and anti‐topoisomerase, in the respective models we assessed those antibodies versus all other patients.

## RESULTS

**Figure 1:**
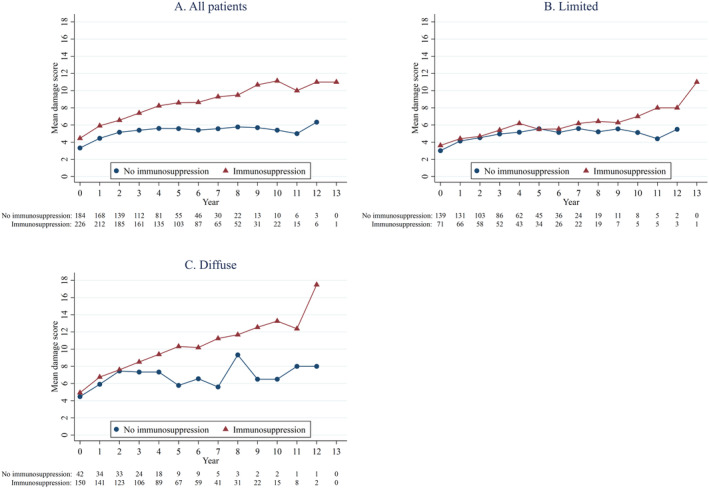
Mean Scleroderma Clinical Trials Consortium Damage Index scores. (A) All patients. (B) Patients with limited cutaneous systemic sclerosis. (C) Patients with diffuse cutaneous systemic sclerosis. All data are from before propensity weighting.

Supplementary Table 1 shows the ISs received at or before the baseline visit. Two patients received both MTX and MPA, so a total of 170 of 402 patients (42%) received some IS already at or before the baseline visit in a cohort of patients with SSc with a disease duration two or fewer years. Figure 1 shows the mean SCTC‐DI values in each group over time. These figures represent unweighted changes, and it can be seen that patients with dcSSc who were immunosuppressed accrued damage at a faster rate than patients who were not immunosuppressed (Figure 1C); in patients with lcSSc, the rate of damage accrual is similar in both patients who were and were not immunosuppressed until about six years, after which patients who were immunosuppressed accrued damage faster (Figure 1B); however, the number of patients is small after that time point.

### Cohort with lcSSc

There were 210 patients in the subset with lcSSc, of which 34% were exposed to ISs at some time (Table 1). Mean (±SD) follow‐up was 4.3 (±2.9) years. Patients who had exposure at some point had higher mean mRSS (5.9 ± 4.3 vs 4.4 ± 4.2; *P* = 0.003), were more likely to have ILD (32.4% vs 19.4%; *P* = 0.037), were more likely to have inflammatory arthritis, were less likely to have anti‐centromere antibody (30.0% vs 59.4%; *P* < 0.001), and were more likely to have anti‐topoisomerase antibodies (33.3% vs 9.7%; *P* < 0.001). Very few patients received ISs only before baseline. The balance diagnostics of the covariates at baseline visit for patients with lcSSc after propensity scoring are illustrated in Supplementary Table 2. The balancing of all variables was better after IPTW, but the SMDs were suboptimal for disease duration and IS before baseline.

Table 2 shows the association between exposure to immunosuppression and the change of damage scores adjusted for possible confounders. Exposure to ISs was associated with lower damage scores. The coefficient for the effect of immunosuppression on the damage index is −0.34 after IPTW (Table 2). A coefficient of −0.34 means that the change in damage score from the previous visit will be 0.34 points less than the change when the patient did not receive ISs. If we take the values from Figure 1B as an estimate of change over time in patients with lcSSc who did not receive ISs, the mean change in score between yearly visits is about 0.3. So, to reduce those scores by 0.34 suggests that the effect of receiving ISs might be substantial. Lower FVC values were associated with more damage accrual, both with and without IPTW.

**Table 2:** Marginal structural GEE model using IPTW to assess the association between exposure to immunosuppression and the change of damage scores, adjusted for potential confounders in patients with limited cutaneous scleroderma*

Characteristic	Without IPTW		With IPTW	
	beta (95% CI)	*P* value	beta (95% CI)	*P* value
Exposure to immunosuppressive drugs	−0.22 (−0.40 to −0.04)	0.019	−0.34 (−0.59 to −0.09)	0.007
Female	−0.16 (−0.40 to 0.07)	0.177	−0.16 (−0.37 to 0.05)	0.144
Age, y	0.004 (−0.004 to 0.01)	0.319	0.001 (−0.01 to 0.01)	0.774
Disease duration	−0.07 (−0.27 to 0.13)	0.513	0.09 (−0.12 to 0.30)	0.407
ACA	−0.03 (−0.32 to 0.25)	0.821	0.01 (−0.26 to 0.27)	0.969
ATA	−0.08 (−0.29 to 0.13)	0.452	−0.13 (−0.34 to 0.08)	0.219
Immunosuppressive drugs before baseline	0.38 (−0.24 to 0.99)	0.230	0.20 (−0.71 to 1.11)	0.661
Damage score at baseline	−0.03 (−0.06 to 0.01)	0.170	−0.002 (−0.04 to 0.04)	0.992
FVC predicted, %	−0.01 (−0.01 to −0.002)	0.008	−0.01 (−0.01 to −0.001)	0.017
CSRG vs ASIG	0.13 (−0.03 to 0.31)	0.118	0.02 (−0.11 to 0.16)	0.738

* ACA, anti‐centromere antibody; ASIG, Australian Scleroderma Interest Group; ATA, anti‐topoisomerase antibody; CI, confidence interval; CSRG, Canadian Scleroderma Research Group; FVC, forced vital capacity; GEE, generalized estimating equation; IPTW, inverse probability of treatment weighting.

### Cohort with dcSSc

There were 192 patients in the subset with dcSSc, of whom 76% were exposed to ISs at some time (Table 3). Mean (±SD) follow‐up was 4.6 (±3.0) years. Patients who were exposed at some point were younger (50.0 ± 12.1 years versus 55.5 ± 10.7 years; *P* = 0.008), were less likely to have anti‐centromere antibodies (3.0% vs 18%; *P* = 0.001), and were more likely to have anti‐topoisomerase antibodies (33.1% vs 10.3%; *P* = 0.005). Very few patients received ISs only before baseline.

**Table 3:** Baseline characteristics of diffuse cutaneous scleroderma patients (dcSSc) stratified according to exposure to immunosuppression at baseline or during follow−up (*N* = 192)*

	Ever exposed at any visit (*n* = 150)		Never exposed at any visits (*n* = 142)		*P* values
		Missing values		Missing values	
		*N*		*N*	
Age, years (mean ± SD)	50.0 ± 12.1		55.5 ± 10.7		0.008
Female, *N* (%)	110 (73.3%)		32 (76.2%)		0.709
Caucasian, *N* (%)	122 (81.3%)		38 (90.5%)		0.160
Education (>high school), *N* (%)	69 (48.6%)	8	18 (42.9%)	0	0.513
Smoking in the past or currently, N (%)	81 (54.4%)	1	29 (69.0%)	0	0.089
Disease duration, years (mean ± SD)	1.1 ± 0.5		1.1 ± 0.5		0.723
mRSS (0−51) (media, IQR)	23.5 (15−29)	2	20 (16−24)	1	0.296
Interstitial lung disease, N (%)	53 (35.3%)		15 (35.7%)		0.964
FVC, % predicted (mean ± SD)	88.3 ± 17.4	1	90.1 ± 23.3	0	0.585
DLCO, % predicted (mean ± SD)	71.2 ± 20.7	11	73.0 ± 16.1	8	0.639
Tendon friction rubs, *N* (%)	41 (27.3%)		11 (26.2%)		0.883
Inflammatory arthritis, *N* (%)	53 (36.3%)	4	10 (25.0%)	2	0.181
Autoantibodies					
Anti‐centromere, *N* (%)	4 (3.0%)	15	7 (18.0%)	3	0.001
Anti‐topoisomerase, *N* (%)	44 (33.1%)	17	4 (10.3%)	3	0.005
Anti‐RNA polymerase III, *N* (%)	55 (44.0%)	25	16 (44.0%)	6	0.962
C−reactive protein, mg/L (median, IQR)	5 (3−12)	10	4.4 (1.9−9.2)	2	0.110
Damage score at baseline (median, IQR)	5 (2−7)		3 (1−6)		1.02
CSRG patients, *N* (%)	77 (51.3%)		26 (61.9%)		0.225
Immunosuppressants prior to baseline, *N* (%)	14 (9.7%)		6 (12.8%)		0.585

* CSRG, Canadian Scleroderma Research Group; DLCO, diffusing capacity of carbon monoxide; FVC, forced vital capacity; IS, immunosuppressives; mRSS, modified Rodnan skin score.

The balance diagnostics of the covariates at baseline visit for patients with dcSSc after propensity scoring are illustrated in Supplementary Table 3. The balancing of sex, mRSS, arthritis, and immunosuppression before baseline were outside expected values, but balancing was good for other variables and adequate for FVC. Table 4 shows the association between exposure to immunosuppression and the change of damage scores adjusted for possible confounders. Exposure to ISs at any time was not associated with damage scores, but exposure to ISs before baseline and lower FVC values were associated with more damage accrual with IPTW.

**Table 4:** Marginal structural GEE model using IPTW to assess the association between exposure to immunosuppression and the change of damage scores, adjusted for potential confounders in patients with diffuse cutaneous scleroderma*

Characteristic	Without IPTW		With IPTW	
	β (95% CI)	*P* value	β (95% CI)	*P* value
Exposure to immunosuppressive drugs	−0.07 (−0.36 to 0.23)	0.667	−0.08 (−0.41 to 0.25)	0.627
Female	−0.34 (−0.66 to −0.02)	0.040	−0.31 (−0.72 to −0.09)	0.132
Age, y	0.001 (−0.01 to 0.01)	0.911	0.004 (−0.01 to 0.02)	0.525
Disease duration, y	−0.24 (−0.57 to 0.08)	0.144	−0.24 (−0.62 to 0.14)	0.210
ATA	0.003 (−0.30 to 0.31)	0.987	−0.04 (−0.38 to 0.30)	0.826
RNAP	0.13 (−0.20 to 0.46)	0.433	0.01 (−0.38 to 0.40)	0.969
Exposure to immunosuppressive drugs before baseline	0.20 (−0.05 to 0.45)	0.109	0.25 (0.02–0.47)	0.036
Damage score at baseline	−0.01 (−0.05 to 0.02)	0.506	−0.01 (−0.04 to 0.02)	0.449
FVC predicted, %	−0.01 (−0.02 to −0.003)	0.004	−0.01 (−0.02 to −0.002)	0.010
CSRG vs ASIG	0.13 (−0.02 to 0.36)	0.238	0.17 (−0.12 to 0.46)	0.255

* ASIG, Australian Scleroderma Interest Group; ATA, anti‐topoisomerase antibody; CI, confidence interval; CSRG, Canadian Scleroderma Research Group; FVC, forced vital capacity; GEE, generalized estimating equation; IPTW, inverse probability of treatment weighting; RNAP, anti–RNA polymerase III antibody.

## DISCUSSION

To our knowledge, this is the first study that has examined the effect of receiving ISs and damage accrual in patients with SSc using the recently developed SCTC‐DI. Because the cohorts studied were observational, IPTW was used to account for the different aspects of disease that may have encouraged the prescription of ISs and to remove as much bias as possible that these different disease characteristics may have had on the outcome. Because damage accrual is so different in patients with lcSSc and dcSSc, and use of ISs was more frequent in patients with dcSSc, we examined each cutaneous subset of patients separately. As such, we were able to find a positive effect of prevention of damage accrual in the subgroup with lcSSc but not in the subgroup with dcSSc.

IS agents have been prescribed extensively to patients with SSc.^2^, ^16^, ^17^, ^28^ Many recommendations and guidelines suggest prescribing ISs for patients with skin and lung disease,^29^, ^30^, ^31^, ^32^, ^33^, ^34^ and in practice, many scleroderma experts agree and follow these recommendations.^35^, ^36^ Although fibrosis may be the proximate cause of most damage in SSc, there has been some evidence that receiving ISs may prevent fibrosis and thus prevent accrual of damage.^10^, ^11^, ^17^, ^29^, ^37^, ^38^, ^39^, ^40^, ^41^, ^42^, ^43^ Most such studies, however, have concentrated on damage to individual organ systems such as the lung or skin, but the development of the SCTC‐DI has allowed us to determine the effect of receiving ISs on a more global measure of damage.^12^, ^13^, ^14^

There are some important limitations to our study. The SCTC‐DI items were determined to a large extent by expert consensus and weighted against mortality and morbidity. We were aware at the time of creating the SCTC‐DI that not all existing databases would include each item, and we agree that some of the items missing from our databases such as small‐joint contractures are important. Unfortunately, we cannot objectively determine how this might have affected our results.

Any study of the effect of an exposure on an outcome using data from observational cohorts is affected by bias. Propensity scoring, such as IPTW, is a method that allows an investigator to assess the impact of an exposure of interest using observational cohorts, in which randomization is not performed, instead of in a prospective randomized trial. Certain patient characteristics that are a common cause of both the observed exposure and the outcome may confound the relationship under study, leading to an over‐ or underestimation of the true effect. IPTW attempts to correct for these when studying the effect of an exposure in an observational cohort.^44^ Somewhat to our surprise, and only partially in keeping with our hypothesis that IS use would prevent damage accrual, we found that IS use prevented damage accrual only in the subset with lcSSc. It is important therefore to try to understand whether the absence of an effect in patients with dcSSc was a true finding or if IPTW was not sufficient to adjust for the biases inherent in using observational cohort data.

We considered several possibilities that may have prevented us from finding a true effect on damage accrual in patients with dcSSc. One is that patients with dcSSc already have considerably more damage within the first two years of disease than those with lcSSc, and therefore, the chance of developing more damage might seem to be less, and thus, it would be more difficult to show prevention of damage accrual. We have found, however, that although the damage index is higher in patients with dcSSc than in those with lcSSc very early in disease progression, in fact, patients with dcSSc continue to accrue damage over subsequent years at a faster rate than patients with lcSSc.^12^, ^13^ Perhaps ISs are just not adequate to suppress this faster rate of damage accrual.

It is also possible that the weighting itself in IPTW was not adequate to account for some of the differences between the populations. Assessing the adequacy of the weighting is done by assessing the standardized differences between groups for all baseline characteristics both before and after weighting. Supplementary Tables  2 and 3 compare the difference in means between groups in units of SD. A standardized difference of <10% (<0.1) may be considered a negligible imbalance between groups.^26^ From Supplementary Table 2 use before baseline. However, from Supplementary Table 3, for dcSSc, weighting was not very good for sex, anti‐centromere antibodies, mRSS, and arthritis. This may have contributed to our inability to find an effect of ISs in the group with dcSSc.

As pointed out by Chesnaye et al,^44^ by accounting for any differences in measured baseline characteristics, the propensity score aims to approximate what would have been achieved through randomization in a randomized controlled trial (RCT); ie, pseudorandomization. In contrast to true randomization, the propensity score can only account for measured confounders, not for any unmeasured confounders.^44^ Therefore, an imbalance in some unmeasured confounder may also have played a role in our inability to find an effect of ISs in patients with dcSSc. This may have been true in the current patients with dcSSc, as indicated by the observation that certain characteristics such as more frequent ILD and arthritis, which might be expected to be associated with more use of ISs, were indeed more prevalent in the group of patients with lcSSc who were exposed to ISs but were found in similar proportions in the groups with dcSSc who were and were not exposed to ISs. This suggests that the decision to prescribe ISs to the patients with dcSSc may have been related to an unmeasured confounder in the subset with dcSSc.

Our study also has important strengths. By combining two observational cohorts, we were able to find a large enough number of patients in both subsets with cutaneous SSc to assess our hypotheses. We have also used a now‐accepted technique of studying the effect of an exposure to a treatment in an observational cohort and have found that at least it seems to be effective in one of the subsets with cutaneous SSc. With these caveats in mind, and with our findings of a protective effect of receiving ISs on damage accrual in patients with lcSSc, we feel that the effect of ISs on damage in patients with dcSSc has not yet been ruled out. We anticipate the use of the SCTC‐DI in future RCTs in SSc and hope that those trials will be able to answer more definitively the question of the benefit of ISs in patients with dcSSc for the prevention of damage accrual.

## AUTHOR CONTRIBUTIONS

All authors contributed to at least one of the following manuscript preparation roles: conceptualization AND/OR methodology, software, investigation, formal analysis, data curation, visualization, and validation AND drafting or reviewing/editing the final draft. As corresponding author, Dr Baron confirms that all authors have provided the final approval of the version to be published, and takes responsibility for the affirmations regarding article submission (eg, not under consideration by another journal), the integrity of the data presented, and the statements regarding compliance with institutional review board/Declaration of Helsinki requirements.

## Supporting information


**Disclosure Form**:

**Supplemental Table 1** Immunosuppressants prior to or at baseline.

**Supplemental Table 2** Balance diagnostics of the covariates at baseline visit for limited cutaneous scleroderma patients (lcSSc).

**Supplemental Table 3** Balance diagnostics of the covariates at baseline visit for diffuse cutaneous scleroderma patients (dcSSc).

## APPENDIX A AUSTRALIAN SCLERODERMA INTEREST GROUP

The following are members of the Australian Scleroderma Interest Group who were not named as authors but all of whom collected data for this paper and reviewed the manuscript: Joanne Sahhar, Monash University; Nava Ferdowsi, Victoria, Australia; Kathleen Morrisroe, University of Melbourne; Laura Ross, University of Melbourne; Gene Siew Ngian, University of Melbourne; Jennifer Walker, Flinders University; Janet Roddy, Fiona Stanely Hospital; and Lauren Host, Sir Charles Gairdner Hospital.

## APPENDIX B CANADIAN SCLERODERMA RESEARCH GROUP

The following are members of the Canadian Scleroderma Research Group who were not named as authors but all of whom collected data for this paper and reviewed the manuscript: Mohammed Osman, University of Alberta; Peter Docherty, Moncton Hospital; Paul Fortin, Universite Laval; Marvin Jacob Fritzler, University of Calgary; Genevieve Gyger, McGill University; Alena Ikic, Universite Laval; Niall Jones, University of Alberta; Elzbieta Kaminska, University of Calgary; Maggie Larche, McMaster University; Sophie Ligier, Hopital Maisonneuve‐Rosemont; Ada Man, University of Manitoba; Ariel Masetto, University of Sherbrooke; Janet Pope, Western University; David Robinson, University of Manitoba; and Tatiana Sofia Rodriguez‐Reyna, Instituto Nacional de Ciencias Médicas y Nutrición.
