# Supplementary material for: Immunosuppressive Drugs in Early Systemic Sclerosis and Prevention of Damage Accrual
Source: Arthritis Care Res (Hoboken). 2025 Feb 2;77(5):640–8. doi: 10.1002/acr.25467 (PMC12038219; doi:10.1002/acr.25467)
Supplement: Supplementary file 2 — Supplemental Table 1 Immunosuppressants prior to or at baseline. [file ACR-77-640-s004.docx]

Supplemental Table 1. **Immunosuppressants prior to or at baseline.**

|  | Limited Cutaneous SSC  N=210 | Diffuse Cutaneous SSC  N=192 |
| --- | --- | --- |
| ***Methotrexate*** |  |  |
| Only prior to baseline | 2 (1.0%) | 13 (6.8%) |
| At baseline | 25 (11.9%) | 61 (31.8%) |
| ***Cyclophosphamide*** |  |  |
| Only prior to baseline | 4 (1.9%) | 4 (2.1%) |
| At baseline | 3 (1.4%) | 13 (6.8%) |
| ***Mycophenloate*** |  |  |
| Only prior to baseline | 0 (0%) | 1 (0.5%) |
| At baseline | 5 (2.4%) | 28 (14.6%) |
| ***Azathioprine*** |  |  |
| Only prior to baseline | 4 (1.9%) | 3 (1.6%) |
| At baseline | 4 (1.9%) | 2 (1.0%) |
