# Supplementary material for: Immunosuppressive Drugs in Early Systemic Sclerosis and Prevention of Damage Accrual
Source: Arthritis Care Res (Hoboken). 2025 Feb 2;77(5):640–8. doi: 10.1002/acr.25467 (PMC12038219; doi:10.1002/acr.25467)
Supplement: Supplementary file 3 — Supplemental Table 2 Balance diagnostics of the covariates at baseline visit for limited cutaneous scleroderma patients (lcSSc). [file ACR-77-640-s002.docx]

**Supplemental Table 2.** Balance diagnostics of the covariates at baseline visit for limited cutaneous scleroderma patients (lcSSc).

|  | Standardized mean difference | |
| --- | --- | --- |
|  | Before weighting | After weighting |
| Age | 0.106 | 0.093 |
| Female | 0.247 | 0.061 |
| Disease duration | 0.199 | 0.365 |
| ACA | 0.953 | 0.097 |
| ATA | 0.489 | 0.032 |
| mRSS | 0.404 | 0.079 |
| Arthritis | 0.737 | 0.016 |
| Immunosuppression prior to baseline | 0.443 | 0.217 |
| Damage score | 0.135 | 0.124 |
| FVC | 0.218 | 0.005 |
