# Supplementary material for: Immunosuppressive Drugs in Early Systemic Sclerosis and Prevention of Damage Accrual
Source: Arthritis Care Res (Hoboken). 2025 Feb 2;77(5):640–8. doi: 10.1002/acr.25467 (PMC12038219; doi:10.1002/acr.25467)
Supplement: Supplementary file 4 — Supplemental Table 3 Balance diagnostics of the covariates at baseline visit for diffuse cutaneous scleroderma patients (dcSSc). [file ACR-77-640-s001.docx]

**Supplemental Table 3.** Balance diagnostics of the covariates at baseline visit for diffuse cutaneous scleroderma patients (dcSSc).

|  | Standardized mean difference | |
| --- | --- | --- |
|  | Before weighting | After weighting |
| Age | 0.387 | 0.020 |
| Female | 0.057 | 0.189 |
| Disease duration | 0.002 | 0.079 |
| ATA | 0.247 | 0.016 |
| RNAP | 0.011 | 0.050 |
| mRSS | 0.092 | 0.141 |
| Arthritis | 0.046 | 0.218 |
| Immunosuppression prior to baseline | 0.006 | 0.029 |
| Damage score | 0.072 | 0.001 |
| FVC | 0.145 | 0.136 |
